# Improving Patient Satisfaction and Associated Factors at Outpatient Department in General Hospitals of Central Zone, Tigray, Northern Ethiopia, June 2018-August 2019: Pre- and Postinterventional Study

**DOI:** 10.1155/2023/6685598

**Published:** 2023-11-15

**Authors:** Gebregziabher Kidanemariam, Hailemikael Gebrekidan, Ebud Ayele Dagnazgi, Kahsay Asfaw

**Affiliations:** ^1^Department of Midwifery, Aksum University, Aksum, Ethiopia; ^2^Department of Pediatrics, School of Nursing, Aksum University, Aksum, Ethiopia; ^3^Department of Public Health Nutrition, School of Public Health, Aksum University, Aksum, Ethiopia; ^4^Semema Primary Hospital, Semema, Ethiopia

## Abstract

**Background:**

Typically, the idea of patient satisfaction is employed to evaluate quality. When patients enter hospitals, they have certain demands for treatment. However, patients may become dissatisfied if their requirements or expectations are not met. There is increasing agreement that evaluating hospital services should be based in part on patients' assessments of the quality of treatment they received overall. The aim of this study was to improve patient satisfaction at outpatient department.

**Objective:**

To assess improving of patient satisfaction and associated factors at outpatient department in general hospitals of central zone, Northern Ethiopia, 2019.

**Methods:**

Pre- and post-intervention study was conducted to assess the patient satisfaction at outpatient department in general hospitals of central zone, between June 2018 and April 2019 using systematic random sampling method. Two hundred seventy-five (275) participants were investigated in the preintervention and postintervention study. Data were entered to SPSS version 20. Binary logistic regression was done to test association of factors with the outcome variable with consideration of *p* value of less than 0.05.

**Result:**

In the preintervention period, the patient satisfaction was 54.2%; after providing intervention, the patient satisfaction was increased to 77% in postinterventional study. Respondents who paid for the medical service were 41% less likely satisfied than those who had gotten free services. Participants whose age of 18-27 years were 22% more likely satisfied than whose age were 58 and greater.

**Conclusion:**

The result in this study shows that the patient satisfaction is higher than other studies done in our country. Sex, age, and those who make payment were significantly associated with patient satisfaction. Despite the result, much things are left to be covered to increase satisfaction, so the concerned bodies, including the regional health bureau, woreda health office, and management committee and board, should mobilize the community and give training to the health professionals to make the environment smoother and more comfortable for patients.

## 1. Introduction

Typically, the idea of patient satisfaction is employed to evaluate quality [1]. Given that it provides information on the provider's performance in meeting patient values and expectations—aspects over which the patient is the final arbiter—it is of fundamental relevance as a gauge of the quality of care. Customer satisfaction and opinions of the quality of health services are strongly correlated, making quality one of the key elements influencing satisfaction. As a result, having satisfied customers helps them experience quality, and consequently, having satisfied customers also helps them experience quality [2].

When patients enter hospitals, they do so with a clear need or desire for care. Patients may become dissatisfied if their wants or expectations are not met, though. There is growing agreement that patient perceptions of overall treatment and satisfaction should be used in part to evaluate the quality of hospital services [1]. The ability to delight patients is essential to providing high-quality medical care; otherwise, even the most technically proficient treatment is useless. Generally speaking, a person's level of satisfaction affects whether they seek medical advice, follow through with therapy, and continue to work with healthcare professionals [3]. Furthermore, it is crucial to execute continual improvements in medical settings and plays an increasingly significant role in the movement toward increased provider accountability [4, 5].

When compared to their expectations, patients' satisfaction frequently reflects how they view both the outcome of the healthcare provided and the caregiving process. The client is satisfied, while if the experience falls short of the expectation, client satisfaction is not realized [6, 7]. The current trend in healthcare delivery is to provide “patient-centered” care, which puts the client at the center of the health delivery system. This means that clients' views and assessments of services provided are crucial in providing feedback for improving the quality of care provided [8].

Patient satisfaction survey is the widely accepted technique and crucial metric to evaluate the quality of care. Hospital management plans all over the world now include patient satisfaction measurement as a key component. Some reports claim that the delivery of healthcare must directly address the preferences and needs of the patient and that higher levels of patient satisfaction boost the effectiveness of medical therapy. Additionally, it is acknowledged that patient satisfaction affects how well they adhere to their treatment plan, seek out medical advice, and maintain a relationship with their practitioners [9].

Business process reengineering (BPR), a tool for a thorough analysis and redesign processes in the service delivery, has been used in Ethiopia since 2010 as part of the national effort of socioeconomic civil service reform to the public sector. Establishing institutions that prioritize serving customers, quickly expanding access to healthcare, and improving the standard of care are its goals for the health sector [10]. Ethiopia Federal Ministry of Health has been leading a sector wide reform effort aimed at significantly improving the quality. The aim of this study was to identify the problems, do preintervention to solve them, and finally improve patient satisfaction.

Patients who are dissatisfied with hospital services are more likely to miss visits, disregard medical advice, or forego prescribed treatments [11]. Patients' satisfaction with medical services has been proven to be influenced by a number of things, such as extended wait times, repeated attempts to get hurt by a needle, bruises from phlebotomy, discomfort during phlebotomy operations, and being treated improperly [12, 13]. Additional concerns cited as affecting patients' happiness included inability to locate a different room inside the hospital, the laboratory staff's refusal to accommodate requests in the afternoon, and a lack of waiting areas [14]. Therefore, the aim of the project is to overcome the root causes of the problem then to enhance patient satisfaction.

### 1.1. Problem Identification and Prioritization

The problem of the health institutions was discussed with management and board members of the hospitals. Through brain storming, the problem was raised. Those problems were low converging institutional delivery, shortage of imaging modality, lack of human power (e.g., pharmacy and laboratory), low patient satisfaction at outpatient department, and shortage of waiting area.

*Problem prioritization*: in order to focus in one identified problem, problems were prioritized using prioritization matrix, and different criteria were used to prioritize the problems (Table 1).

Among the selected problems, cases were rated based on the criteria of feasibility, community concern, government concern, and cost effectiveness. Based on these criteria, the one with the highest score was low patient satisfaction at outpatient department.

*Feasibility*: refers to the problem feasible or likely to solve through intervention within a given period of time. The more feasible it is, the more value is given.

*Time*: in this case refers to the problem that can be solved within a short period of time, the problem that can be solved within a short period of time given high number.

*Cost*: refers to the amount of money the problem is going to cost in order to solve, the problem that can be relatively cost less given high number.

*Community concern*: this refers to how much is the problem given attention by the community, the problem which has high attention by government given high number.

*Government concern*: this refers to how much is the problem given attention by the government, the problem which has high attention by government given high number.

## 2. Methods

### 2.1. Study Area and Study Period

This study was carried out at public hospitals of central zone. Central zone is one of the zonal administrations of Tigray which is located approximately 1024 km away from Addis Ababa and 240 kilometers from Mekelle, and Tigrigna language is spoken in the zone. The total population of the zone by population projection from zonal 2018 official report is 1,450,799 of which about 739,907 are female population. Administratively, it is divided into 22 districts, and it has three general hospitals. The hospitals provide inpatient and outpatient services, including maternal and child care. The pre- and postintervention was conducted from June 2018 to April 2019.

### 2.2. Study Design

Institution-based pre- and postinterventional study was conducted to evaluate the patient satisfaction.

### 2.3. Source Population

The source population was all adults aged 18 years and above and patients visiting the outpatient department in the health institutions.

### 2.4. Study Population

Those who were seen and treated in the outpatient department during study period were the study population.

### 2.5. Inclusion and Exclusion Criteria

#### 2.5.1. Inclusion Criteria

The inclusion criteria are as follows: all outpatient department patients aged ≥18 years and those who have willingness to participate in the study during study period.

#### 2.5.2. Exclusion Criteria

The exclusion criteria are as follows: patients aged ≥18 years, unable to speak, and seriously ill.

### 2.6. Sample Size Determination

The actual sample size was calculated using a single population proportion formula. By considering the following assumptions, 95% confidence level, where *Z* *α*/2 = 1.96, *p* is the previous percentage level of outpatient satisfaction (22%) (20), and *d* = 5%, margin of error will be taken. Therefore,*n* = 263, and adding the nonresponse rate of 5%, therefore, the final sample size was 275.

### 2.7. Sampling Technique

In general hospitals, monthly patient flow reviewed from the document was 2460. Through sample size calculation, a total of 275 patients were needed. Data were collected for one month, and systematical random sampling method was used.

### 2.8. Description of the Intervention

The root causes for low outpatient satisfaction were long waiting time, lack of triage service, and poor patient handling. The following alternative interventions were selected:

1. Increasing the number of outpatient department rooms through arranging the existing rooms
2. Introducing triage service
3. Establishing patient appointment system
4. Training of staff on how to handle patients and service provision
5. Setting rules to staff for patient satisfaction and cares

#### 2.8.1. Selection of the Best Interventions

The best interventions are training of staff on how to handle patients and service provision, introducing triage service, and increasing the number of outpatient department rooms through arranging the existing room (Table 2).

#### 2.8.2. Types of Indicators

The outcome indicator (primary) is the percentage of outpatient satisfaction.

The process indicators (secondary) are the percentage of outpatient department staff trained, availability of triage, average waiting time at the outpatient department, and the number of outpatient department rooms increased through arranging the existing rooms.

### 2.9. Data Collection Method (Variables) Indicator-Based Description

A structured pretested questionnaire was prepared via reviewing of literatures. The questionnaire was initially prepared in English and translated into Tigrigna (local language) and again was retranslated back to English to check for any inconsistencies or distortions in the meaning of words and concepts. Face-to-face interview was the technique of data collection. For administering the interview, data collectors were recruited and trained.

### 2.10. Operational Definition

#### 2.10.1. Patient Satisfaction

Together, the five measurement components of the satisfaction scale produce a maximum score of 110 and a lowest score of 22. The responses were summed up, and a total score was obtained for each respondent. Mean score was computed, and those who are above mean are categorized as satisfied whereas those who score below the mean are categorized as unsatisfied.

#### 2.10.2. Outpatient Department

The outpatient department is the location where routine patients receive follow-up care, except for the emergency room and the mother and child health department.

### 2.11. Data Quality Control

Qualities of data were controlled through training of data collectors, conducting pretest, close supervision, and follow-up of the filled questionnaire. Pretest was done one week before the actual data collection among 14 samples (5%) from outpatient department of Suhul General Hospital, 42 km far from central zone. During pretesting, the questionnaires were checked for its clarity, simplicity, understandability, completeness, consistency, and coherency. The principal investigator strictly supervises the data collection process and provides on-site advice as required. Data collectors were checked by supervisors on daily basis. The daily task was observed intently, and it was promptly evaluated and given feedback.. Daily information exchange including by telephone was a means used to correct problems during the course of the data collection.

### 2.12. Data Analysis

Baseline situational assessment was conducted to measure the magnitude of patient satisfaction and to identify factors contributing to its occurrence post to the intervention. The appropriate strategy was implemented as an intervention. Data was entered and analyzed using SPSS software version 23. Satisfaction of pre- and postintervention was computed using the Likert scale measurement. Respondent who scores greater than the mean was considered satisfied in each question. Descriptive and binary logistic regression analysis was done to identify factors which could affect level of satisfaction by consideration 95% CI and *p* value less than 0.05.

### 2.13. Interventions

According to the comparative analysis of interventions, best interventions like training on how to handle patients, introducing triage, and increasing the number of outpatient department rooms through arranging the existing rooms were selected to improve the patient satisfaction at outpatient department of central zone general hospitals. Outpatient health providers received three days of training on how to treat patients with care, respect, compassion, and quality and equity services, including the six dimensions of quality service like patient-centered care, safety, equity, timely, effectiveness, and efficiency, to improve their knowledge.

Through close communication with management body of the hospital, the importance to have a triage service in order to solve patient satisfaction, finally through this discussion on agreement, was reached. One room was arranged, and the service was beginning to serve for the patients. To solve the long waiting time in outpatient department in addition to the above intervention, room arrangement for outpatient department was conducted before the intervention; there were two adult outpatient departments through discussion with the management body of the hospital; the number of outpatient departments increased into three; this helps to reduce the outpatient department waiting time from 40 min to 30 minutes.

## 3. Results

### 3.1. Sociodemographic Characteristics

The total number of participants during preintervention and postintervention was 275, and the response rate was 100%. Among study subjects, the majority of participants during preintervention were within age range of 38-47 (26.7%) which was quite similar to postinterventional respondents age 38-47 (27.6%) (Table 3).

### 3.2. Medical Service-Related Data

During preintervention, the majority had been paid to get services (144, 52.5%) which was again similar in the postinterventional, and the majority had been paid to get services (146, 53.1%) (Table 4).

### 3.3. Satisfaction Level of Respondents

The satisfaction level of respondents was measured based on different angles. After preintervention study, out of 275 participants, 149 (54.2%) of them were satisfied, and after intervention, 275 respondents were assessed; about 212 (77%) of them were satisfied (Table 5).

### 3.4. Associated Factors of Status of Satisfaction

Binary logistic regression was done to assess association of independent variables with the outcome variable with consideration of 95% confidence interval and *p* value of less than 5%. Variables which were significant at bivariate logistic regression with *p* value of less than 0.25 were included in the multivariate logistic regression. Age, sex, educational status, payment, and occupation were significant, but after multivariate logistic regression, employed age, payment status, and sex were significant at bivariate logistic regression.

In multivariate logistic regression, respondents who paid for the medical service were 41% (AOR = 0.59, 95% CI: 0.11, 0.92) less likely satisfied than those who had gotten free services. Participants whose age of 18-27 years were 22% (AOR = 1.22, 95% CI: 1.082, 14.3) more likely satisfied than whose age were 58 and greater. Male respondents were 5.8 times (AOR = 5.8, 95% CI: 1.8, 15.2) more likely satisfied than females (Table 6).

## 4. Discussion

In the preintervention period, the overall participant satisfaction (54.2%) was less than the postinterventional assessment period which was 77%. This improvement in satisfaction might be related to intervention actions that included training, introducing triage, and increasing outpatient department rooms though arranging from existing rooms.

Patients' satisfaction with medical services has been proven to be influenced by a number of things, such as extended wait times, repeated attempts to get hurt by a needle, bruises from phlebotomy, discomfort during phlebotomy operations, and being treated improperly [6, 7]. As additional factors affecting patients' satisfaction, the inability to locate a different room within the hospital, the laboratory staff's refusal to accommodate requests in the afternoon, and the lack of waiting areas were mentioned. However, this study's association variables were sex, age, and payment status, which differs from the study conducted in Addis Ababa [14]. This might be due to the fact that here the study is postinterventional.

Patient satisfaction levels in Ethiopia's outpatient departments of various hospitals ranged from 22.0% in Gondar to 57.1% in Jimma, according to several studies [15, 16]. The study done here is much higher than that of Gondar but a bit higher than of Jimma. This difference might be the study design and seasonal variation.

The study participants with age range of 18-27 were more satisfied than the older. This is similar to the study finding in Tikur Ambesa, whose of this age (18-30) had higher satisfaction than older participants [17].

Eastern Ethiopia reported that there was a substantial correlation between patient address and responder payment status and patient satisfaction with healthcare [18], which is comparable to this research. This can be the result of similar economic and cultural ideals.

Generally, as overall, the satisfaction rate of respondents was higher in the postintervention study which was inclined from 54.2% to 77%. Satisfaction on pharmacy technician during dispensing drug was higher in the postintervention which was inclined from 52.2% to 80%. This could be due to the training given to the hospital staff and over commitment of the professionals. Satisfaction on examination room was higher in the postintervention study which was raised from 48.1% to 78.2%. This is again probably due to the special orientation given to the hospital community that they make it clean, getting awareness of how to keep the privacy of patient in the examination room, and increase OPD number from 2 to3.

Satisfaction towards water access was higher in the postintervention period which was raised from 49.4% to 76.3; satisfaction to latrine neatness was higher in the postintervention study which was increased from 49.2% to78.1%. This could be probably due to the training given to health providers to provide water accessibility and the hospital janitors to make clean and safe latrines which are found in the hospitals.

Satisfaction towards respecting patients was higher in the postintervention study which was raised from 53.1% to 77.4%. This is again due to the fact that the clinicians were fully trained on how to handle their patients during emergency and in usual time could be due to the motivation of the staff in the postintervention time. Satisfaction of participants towards vital sign measurement in the postintervention study was higher than in the preintervention study which was increased from 60.8% to 76.7%. This might be due the professional variation from time to time and the nature of the disease.

Satisfaction towards knowing their cases was increased in the preintervention and postintervention from 57.1% to 77.8%, respectively. In this study, patient's satisfaction on working hour during postintervention (78.1%) was higher than during preintervention time. This might be because most of the hospital staff members attended on time and staffs were wasting their time on work activities.

## 5. Conclusion

As the result of the intervention on job training, regular patient satisfaction measurement and increasing of number of OPD rooms through arranging of the existing rooms lead to the patient satisfaction at OPD in general hospitals to increase from 54.2% to 77%.

The status of satisfaction in this study was higher than other studies conducted Ethiopia. Age, payment, and sex of participants were found significant. Lastly, the hospitals should continue training their staff on care, respect, compassion, and quality of service. It should also perform periodic evaluations of clients' satisfaction.

## 6. Limitation of the Study

Due to the short duration required to make the proper intervention, the postintervention result was not such satisfactory. It would have been better if the study conducted in qualitative approach in order to investigate the depth overview of the clients.

## Figures and Tables

**Table 1:** Problem prioritization matrix at general hospitals of central zone, Tigray, Northern Ethiopia, 2018.

S. no.	Identified problems	Feasibility	Time	Cost	Community concern	Government concern	Total	Rank
1	Shortage of imaging modality	1	1	2	4	3	11	4
2	Shortage of human power (e.g., pharmacy and laboratory) technical	2	1	2	4	3	12	3
3	Low patient satisfaction at OPD	3	3	3	4	4	17	1
4	Low converging institutional delivery	2	2	3	3	5	15	2
5	Shortage of waiting area	1	1	1	4	3	10	5

NB. The criteria's value ranges from 1 up to 5 and given by senior management team of the hospital based on the criteria.

**Table 2:** Comparative analysis of alternative interventions using criteria.

Strategy alternatives	Impact	Cost	Time	Feasibility	Total score	Rank
Establishing appointment system	1	5	5	2	13	4
Training of staff on how to handle patients and service provision	5	3	4	5	17	1
Setting rules to staff for patient satisfaction and cars	2	5	5	3	15	3
Introducing triage service	5	3	4	4	16	2
Increasing the number of outpatient department rooms through arranging the existing rooms	5	4	4	3	16	2

**Table 3:** Sociodemographic characteristics among respondents of general hospitals of central zone of Tigray, Northern Ethiopia, 2019.

Preintervention (*N* = 275)		Postintervention (*N* = 275)
Variables	Freq (%)	Freq (%)
Age		
18-27	57 (20.8)	58 (21.1)
28-37	65 (23.6)	69 (25.1)
38-47	73 (26.7)	76 (27.6)
48-57	54 (19.7)	52 (18.9)
≥58	26 (9.2)	20 (7.3)
Sex		
Male	155 (56.4)	150 (54.5)
Female	120 (43.6)	125 (45.5)
Marital status		
Unmarried	70 (25.5)	74 (26.9)
Married	150 (54.5)	158 (57.5)
Separated	30 (10.9)	25 (9.1)
Widowed	25 (9.1)	18 (6.5)
Educational status		
Illiterate	55 (20)	65 (23.6)
Able to read and write	130 (47.3)	101 (36.7)
Primary school	35 (10.9)	31 (11.3)
Secondary school	40 (14.5)	55 (20)
Diploma and above	15 (5.5)	23 (8.4)
Ethnicity		
Tigray	271 (98.5)	273 (99.3)
Amhara	4 (1.5)	2 (0.7)
Religion		
Orthodox	247 (89.8)	252 (91.6)
Muslim	28 (11.2)	23 (8.4)
Occupation		
Civil servant	36 (13.1)	32 (11.6)
Private	36 (13.1)	32 (11.6)
Merchant	54 (19.6)	52 (18.9)
Farmer	123 (44.7)	132 (48)
Unemployed	5 (1.8)	3 (1.1)
Student	21 (7.6)	24 (8.7)
Residence		
Urban	80 (29.1)	94 (34.2)
Rural	195 (70.9)	181 (65.8)

**Table 4:** Medical service-related data among respondents of general hospitals of central zone of Tigray, Northern Ethiopia, 2019.

Preintervention		Postintervention
Variables	Freq (%)	Freq (%)
Payment status		
Free	131 (47.6)	129 (46.9)
Pay	144 (52.4)	146 (53.1)
Number of visits		
New	90 (32.7)	84 (30.5)
Repeated	177 (64.4)	183 (66.5)
3^rd^	3 (1.1)	3 (1.1)
4^th^	5 (1.8)	5 (1.8)
Lab. order		
Yes	130 (47.3)	115 (41.8)
No	145 (52.7)	160 (58.2)
Waiting time for lab (*n* = 130)		*N* = 115
<1 hours	100 (76.9)	67 (58.3)
1-2 hours	20 (15.4)	39 (33.9)
>2 hours	10 (6.7)	9 (7.8)
Medication order		
Yes	258 (93.8)	263 (95.6)
No	17 (6.2)	12 (4.4)
Medication available in pharmacy		
Yes all	210 (76.4)	250 (90.9)
Half	20 (7.3)	11 (4)
Less than half	15 (5.5)	8 (2.9)
No	30 (10.8)	6 (2.2)

**Table 5:** Satisfaction of respondents in pre- and postintervention period of general hospitals of central zone of Tigray, Northern Ethiopia, 2019.

Pre intervention (*N* = 275)			Postintervention (*N* = 275)	
Variables	Satisfied freq (%)	Dissatisfied freq (%)	Satisfied freq (%)	Dissatisfied freq (%)
Satisfaction on working hours	165 (60%)	110 (40%)	215 (78.1)	60 (21.9)
Satisfaction waiting time	151 (55%)	124 (45%)	210 (76.3)	65 (23.7)
Satisfaction on quality of services	176 (63.9)	99 (36.1)	220 (80)	55 (20)
Satisfaction on expected needs	167 (60.6)	108 (39.2)	213 (77.4)	62 (22.6)
Satisfaction on medication access	157 (57.2)	118 (42.8)	200 (72.7)	75 (27.3)
Satisfaction on medical equipment access	159 (57.8)	116 (42.2)	230 (83.6)	45 (16.4)
Satisfaction on waiting area	138 (50.1)	137 (49.9)	215 (78.1)	60 (21.9)
Satisfaction on clinician support	153 (55.6)	122 (44.4)	220 (80)	55 (20)
Satisfaction on competency of health professionals	163 (59.4)	112 (40.6)	200 (72.7)	75 (27.3)
Satisfaction on health professional access	154 (56.1)	121 (43.9)	225 (81.8)	50 (18.2)
Satisfaction on medical equipment quality	180 (65.3)	95 (34.7)	217 (78.9)	58 (21.1)
Satisfaction on pharmacy technician during dispense of drug	144 (52.2)	131 (47.8)	220 (80)	55 (20)
Satisfaction on examination room	132 (48.1)	143 (51.9)	214 (77.8)	61 (22.2)
Satisfaction on chair for sitting	126 (45.8)	149 (54.2)	216 (78.5)	59 (21.5)
Satisfaction on water access	136 (49.4)	139 (50.6)	210 (76.3)	65 (23.7)
Satisfaction on clean latrine	135 (49.2)	140 (50.8)	215 (78.1)	60 (21.9)
Satisfaction on direction indicator	153 (55.8)	122 (44.2)	200 (72.7)	75 (27.3)
Satisfaction hospital ventilation	141 (51.4)	134 (48.6)	220 (80)	55 (20)
Satisfaction on respecting and support of professionals	146 (53.1)	129 (46.9)	213 (77.4)	62 (22.6)
Satisfaction on vital sign measurement	167 (60.8)	108 (39.2)	211 (76.7)	64 (23.3)
Satisfaction of explaining their disease	159 (57.8)	116 (42.2)	214 (77.8)	61 (22.2)

**Table 6:** Predicators of patient satisfaction in general hospitals of central zone of Tigray, Northern Ethiopia, 2019.

Variables	Status of satisfaction		COR (95% CI)	AOR (95% CI)	*p* value
	Satisfied	Dissatisfied			
Payment status					
Pay	76 (58.9%)	53 (41.1%)	0.66 (0.123-0.476)	0.59 (0.109-0.918)	0.045^∗^
Free	100 (68.5)	46 (31.5%)			
Sex					
Male	92 (61.3%)	58 (38.7%)	0.77 (0.485-0.803)	5.8 (1.80-15.15)	0.023^∗^
Female	84 (67.2%)	41 (32.8%)			
Age category					
18-27	41 (70.7%)	17 (29%)	1.034 (1.24-1.98)	1.22 (1.082-14.26)	0.034^∗^
28-37	41 (59.4%)	28 (40.6%)	0.628 (1.876-4.761)	2.1 (1.8-18.6)	
38-47	49 (64.5%)	27 (35.5%)	0.778 (0.678-0987)	3.04 (2.98-25.87)	
48-57	31 (59.6%)	21 (40.4%)	0.633 (0.123-0.347)	5.8 (4.99-16.6)	
≥58	14 (70%)	6 (30%)	—	—	

## Data Availability

The data used to support the findings of this study are included in the article.

## Abbreviations

BPR: Business process reengineering
CEO: Chief executive officer
CI: Confidence interval
CRC: Care respectful compassion
OPD: Outpatient department
SPSS: Statistics Package for Social Sciences
TQM: Total quality management
WHO: World Health Organization.
